# Estimation of Muscle Forces of Lower Limbs Based on CNN–LSTM Neural Network and Wearable Sensor System

**DOI:** 10.3390/s24031032

**Published:** 2024-02-05

**Authors:** Kun Liu, Yong Liu, Shuo Ji, Chi Gao, Jun Fu

**Affiliations:** School of Mechanical and Aerospace Engineering, Jilin University, Changchun 130000, China; liuyong19@mails.jlu.edu.cn (Y.L.); jishuo20@mails.jlu.edu.cn (S.J.); gaochi21@mails.jlu.edu.cn (C.G.); fujun21@mails.jlu.edu.cn (J.F.)

**Keywords:** muscle forces, neural network, inertial sensor, CNN–LSTM

## Abstract

Estimation of vivo muscle forces during human motion is important for understanding human motion control mechanisms and joint mechanics. This paper combined the advantages of the convolutional neural network (CNN) and long-short-term memory (LSTM) and proposed a novel muscle force estimation method based on CNN–LSTM. A wearable sensor system was also developed to collect the angles and angular velocities of the hip, knee, and ankle joints in the sagittal plane during walking, and the collected kinematic data were used as the input for the neural network model. In this paper, the muscle forces calculated using OpenSim based on the Static Optimization (SO) method were used as the standard value to train the neural network model. Four lower limb muscles of the left leg, including gluteus maximus (GM), rectus femoris (RF), gastrocnemius (GAST), and soleus (SOL), were selected as the studying objects in this paper. The experiment results showed that compared to the standard CNN and the standard LSTM, the CNN–LSTM performed better in muscle forces estimation under slow (1.2 m/s), medium (1.5 m/s), and fast walking speeds (1.8 m/s). The average correlation coefficients between true and estimated values of four muscle forces under slow, medium, and fast walking speeds were 0.9801, 0.9829, and 0.9809, respectively. The average correlation coefficients had smaller fluctuations under different walking speeds, which indicated that the model had good robustness. The external testing experiment showed that the CNN–LSTM also had good generalization. The model performed well when the estimated object was not included in the training sample. This article proposed a convenient method for estimating muscle forces, which could provide theoretical assistance for the quantitative analysis of human motion and muscle injury. The method has established the relationship between joint kinematic signals and muscle forces during walking based on a neural network model; compared to the SO method to calculate muscle forces in OpenSim, it is more convenient and efficient in clinical analysis or engineering applications.

## 1. Introduction

Human movement is an external manifestation of the neuromuscular skeletal system [1]. Estimating muscle forces is an important research direction in studying human motion control mechanisms and understanding joint mechanics. Furthermore, estimating muscle forces is essential in many research fields, including quantitative assessment of the contribution of different muscles in human motion [2], quantitative evaluation of muscle injury and evaluation of rehabilitation training effectiveness [3,4], and providing theoretical support for the design of rehabilitation equipment, such as prostheses, orthotics, and exoskeletons [5,6,7,8].

Although estimating muscle forces has important theoretical and practical significance, there are still significant challenges in estimating muscle forces during dynamic movements [9]. A commonly used method for estimating muscle forces is to use a rigid musculoskeletal model despite its limitations [10,11]. It is obvious that for each joint in the human body, its own degree of freedom is always smaller than the number of muscles participating in the joint movement, which makes it difficult to estimate muscle forces using a rigid musculoskeletal model. To solve the redundancy problem, static optimization (SO) is usually adopted to estimate the muscle forces by optimizing several objective functions, such as minimization of the muscle activation squared [12,13]. However, the optimization process itself requires high computational power, and as the number of muscles involved in the optimization process increases, the complexity of the optimization process also increases. Recently, biomechanical analysis software has been established to analyze human motion, such as OpenSim [14,15]. The software provides a model-based estimation method for establishing musculoskeletal models and analyzing human motion and muscle function. When calculating muscle forces using OpenSim, the static optimization calculation process can be simulated in the software, which greatly simplifies the complexity of muscle force calculations. However, the OpenSim-based muscle force calculation relies on professional experimental equipment, including the optical motion capture system (OMCS) and force platform; therefore, the application of the method is greatly limited to scenarios outside the laboratory environment.

Given the limitations of using a rigid musculoskeletal model to estimate muscle forces, more and more researchers are focusing their research on the relationship between surface electromyography (sEMG) signals and muscle forces. The sEMG signals are electrical signals accompanying muscle contraction and are considered a reliable method for detecting muscle activity. Many computational algorithms were established to reveal the sEMG–force relationship during the last several decades, including the Hill model [16], polynomial fitting model [17], fast orthogonal search (FOS) [18], and parallel cascade identification (PCI) [19]. In recent years, with the rapid development of computer technology, artificial intelligence has been applied in a wide range of fields, and many studies have applied neural networks to muscle force estimation based on the sEMG. Hua et al. combined linear regression (LR) and LSTM to estimate muscle forces from sEMG signals [20], and the experiment results showed that the LR–LSTM performed well in muscle force estimation. Xu et al. compared the performance of CNN, LSTM, and CNN–LSTM in estimating muscle forces from sEMG signals [21], and the experiment results showed that CNN–LSTM performed best. Choi et al. built a fully connected neural network, and the performance of the model was satisfactory when it was trained to estimate the palmar pinch force [22]. Peng et al. proposed a time-delayed neural network (TDNN) for estimating muscle forces during shoulder isometric contraction, and the model was less prone to overfitting and had better robustness compared to commonly used feedforward networks [23]. Jie et al. estimated the muscle forces of the biceps femoris short head (BFS) and the rectus femoris (RF) using a CNN network based on the sEMG [24]. Hu et al. estimated the grasping forces using an LSTM network from high-density surface EMGs [25]. Although many achievements have been made in estimating muscle forces using neural network models and sEMG, the collection and processing of sEMG have always been a hindrance to its widespread application. The sEMG itself is a weak electrical signal that is highly susceptible to external interference, so frequent calibration is required before each task of collecting the signals. In addition, only the sEMG of superficial muscles can be measured, and it is difficult to measure the sEMG of muscles covered by superficial muscles.

Compared to sEMG signals, wearable inertial sensors have the advantages of small size, low cost, and ease of wear, making it very convenient to collect human gait motion information. Tobias analyzed gait symmetry based on a wearable inertial sensor [26]. Debin proposed an attention-based freezing of gait prediction method based on wearable inertial signals and a deep neural network model; the method provides a new potential for PD patients’ fall prevention in daily life [27]. Brognara investigated the fall risk among patients with diabetic foot neuropathy using wearable inertial sensors [28]. Yun achieved new dynamic muscle force estimation by utilizing a wearable ultrasound transducer and an IMU sensor [29]. However, few studies have combined neural network models with wearable inertial sensors to estimate muscle forces. Tien estimated the muscle forces of rectus femoris (RF), soleus (SOL), and tibialis anterior (TA) based on the LSTM network, but the kinematic data required to train the neural network model was retrieved from a public repertory [30]. Suin et al. developed a wearable sensor system and estimated the lower limb muscle forces; however, the estimation method was an inverse dynamics-based static optimization method [31]. We also discussed gait phase recognition using DPF–LSTM–CNN [32] and muscle force estimation in the STS process using sensors [33].

In this study, we have combined the neural network and the wearable sensor system to achieve an estimation of lower limb muscle forces during walking. Firstly, we have developed a wearable sensor system, including six inertial sensors, which were used to collect the ankle, knee, and hip joint angles and joint angular velocities in the sagittal plane, and the accuracy of the sensor system was verified. Secondly, we have proposed the CNN–LSTM model by combining CNN and LSTM, which was expected to have both the advantages of CNN for feature extraction and the advantages of LSTM for temporal sequence data processing. Finally, we conducted experimental verification on the performance of CNN–LSTM in muscle force estimation.

## 2. Materials and Methods

### 2.1. Calculation of Muscle Force

OpenSim was used to calculate the lower limb muscle forces in this paper, where the data collected by the OMCS and force platform were used to scale a generic musculoskeletal model for each subject. Then, the joint kinematics parameters of each subject could be obtained by the inverse kinematic (IK) tool for static optimization (SO) to calculate the muscle forces. The calculated results were considered as the reference values compared with the muscle forces estimated by the neural network model. The generic musculoskeletal model used in this paper was the Gait2392 model provided by OpenSim, which included 23 bones, 92 muscles, and 39 external markers. In this paper, the gluteus maximus (GM), rectus femoris (RF), gastrocnemius (GAST), and soleus (SOL) of the left leg were selected as the research target muscles. At different walking speeds, the above four muscles play an important role in the human body’s walking process [34].

### 2.2. Neural Network for Muscle Forces Estimation

In this paper, the advantages of CNN and LSTM were combined to propose a CNN–LSTM neural network model for estimating lower limb muscle forces during human walking. To evaluate the performance of CNN–LSTM, the standard CNN and LSTM were also used to estimate muscle forces, and their estimation results were compared with those of CNN–LSTM.

#### 2.2.1. Structure of Standard CNN

CNN has extensive applications in fields such as computer vision, speech recognition, and natural language processing [35,36,37]. The convolution layer and pooling layer are the two most important parts of CNN. Convolutional layers are used for extracting data features while pooling layers are used to reduce the number of parameters in the model and prevent overfitting. Equation (1) is the mathematical expression for the output of the convolutional layer, where *x_j_^l^*, and *x_i_^l^*^−1^ are the output data feature and input data feature of the *l*-layer, respectively; *W_j_^l^*, and *b_j_^l^* represent the convolution kernel weight and the corresponding offset, respectively; *f* represents the activation function; and ReLU was selected in this paper.

(1) $$x_{j}^{l}=f\left(\sum_{i=1}^{M}\left(x_{i}^{l-1}\times W_{j}^{l}\right)+b_{j}^{l}\right)$$

Following each convolution, pooling is usually carried out to reduce overfitting and increase the network’s receptive field size. Max-pooling is a commonly used pooling operation that reduces the amount of data by maximizing it [38]. The usual operation is to divide the input image into several rectangular regions and output the maximum value for each subdomain. In this paper, the input sequence data were reshaped to a rectangle image when applying CNN to the muscle forces estimation. Then, a series of convolutions were carried out, followed by max-pooling. The outcome was encoded and flattened by a fully connected layer to output the estimated muscle forces. There are two convolution layers with 64 and 128 convolutional kernels in CNN. The size of the convolution kernels was 3 × 1, and the convolution step was 1. The size of the pool group in the two layers of pooling layer was 2 × 1, the step size of the first layer of pooling layer was 1, and the step size of the second layer of pooling layer was 1.

#### 2.2.2. Structure of Standard LSTM

LSTM, as an improved version of recurrent neural network (RNN), mainly solves the problems of gradient disappearance and gradient explosion when RNN is used to process the long time series. In short, LSTM can perform better in longer time series than ordinary RNN. Compared with traditional RNN, LSTM units have added forgetting gate, update gate, and output gate, which is why LSTM can solve the long-term dependency problem of RNN. The functions of the three gates mentioned above can be summarized as: the forgetting gate determines the retention and discarding of information in old storage units, update gate determines whether the information needs to be stored in the current storage unit, and output gate controls the output based on the input and storage units. The detailed calculation of the LSTM unit can be easily found in related works, so the specific calculation process will not be shown in this paper. LSTM is employed in this work with double layers. The two hidden layers contain 128 units, and the activation function used by the hidden layer is the tanh function.

#### 2.2.3. Structure of CNN–LSTM

**Figure 1:**
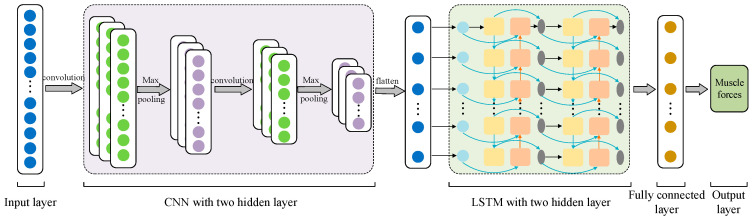
Structure of convolutional neural network–long-short-term memory CNN–LSTM.

Based on the above-introduced neural network structure for estimating lower limb muscle forces during gait using CNN and LSTM separately, a CNN–LSTM could be easily attained by simply stacking convolution layers and LSTM layers. In the process of using CNN–LSTM, the input sequence data were fed into a convolution layer firstly, in which the convolution calculation with stride was performed. Then, the extracted higher-level feature map went through an LSTM layer to produce prediction output. Finally, the estimated forces were exported through a fully connected layer. The specific structure of CNN–LSTM is shown in Figure 1. In this paper, the mean absolute error (MAE) loss function was used. The optimization process was based on the efficient Adam stochastic gradient descent method [39].

#### 2.2.4. Evaluation Method

To quantify the estimation performance of the CNN, LSTM, and CNN–LSTM, percentage root mean square error (*RMSE*%) was selected as the comparison metric [19]. It was calculated as follows:

(2) $$RMSE\%=\sqrt{\frac{\sum_{i=1}^{n}{\left(x_{i}-y_{i}\right)}^{2}}{\sum_{i=1}^{n}{\left(x_{i}\right)}^{2}}\times 100\%}$$

where *x_i_* is the actual value, *y_i_* is the predicted value, and *n* is the number of data points.

In addition, Pearson’s correlation coefficient (*r*) was also employed as another comparison metric, which could be calculated as follows:

(3) $$r=\frac{\sum_{i=1}^{n}\left(x_{i}-{\bar{x}}_{i})(y_{i}-{\bar{y}}_{i}\right)}{\sqrt{\sum_{i=1}^{n}{\left(x_{i}-{\bar{x}}_{i}\right)}^{2}}\sqrt{\sum_{i=1}^{n}{\left(y_{i}-{\bar{y}}_{i}\right)}^{2}}}$$

where ${\bar{x}}_{i}$ is the mean of the actual value, ${\bar{y}}_{i}$ is the mean of the predicted value.

## 3. Experiment

### 3.1. Data Collection and Preprocessing

**Figure 2:**
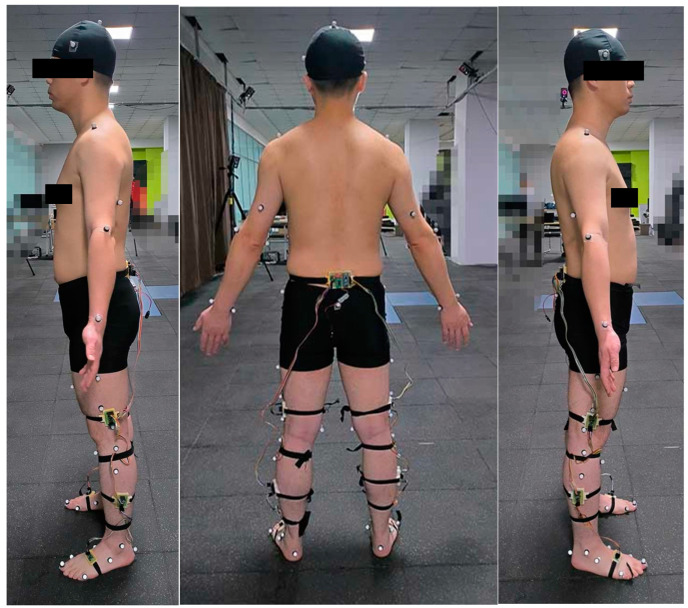
Experiment with the optical motion capture system (OMCS) and the self-developed wearable inertial sensor system.

When using OpenSim to calculate the lower limb muscle forces during gait, it was necessary to establish a personalized musculoskeletal model based on human gait kinematics, dynamics data, and the ground reaction force. In this paper, the gait kinematics and dynamics data were collected by an 11-camera motion capture system (Vicon, Oxford, UK) at a frequency of 1000 Hz, and the ground reaction force was collected by three force plates (AMTI Inc., Watertown, MA, USA) at a frequency of 200 Hz, as shown in Figure 2.

A self-developed wearable inertial sensor system was worn on the subject to collect the lower limb joint angles and joint angular velocities in the sagittal plane during human walking, as shown in Figure 2, and the collected data were used as input for the neural network model. The system mainly included a micro processing unit (Arduino nano), six IMUs (JY901), a wireless transmission module (nrf24l01). When the sensor system was working, the Arduino nano collected the joint angles and joint angular velocities measured by JY901 at a frequency of 100 Hz, then sent the data to the host computer in real-time by nrf24l01. When different individuals performed the same kinematic characteristics, there would still be differences in the strength of lower limb muscles due to the differences in height and weight of the subjects. Therefore, except for the joint angle and angular velocity, the height, weight, thigh length, and shark length were also selected as inputs for the neural network model. Finally, the input feature size of the neural network model was sixteen.

In order to reduce the training complexity of the neural network, the data should be normalized before input to the neural network. The mathematical expression of data normalization was as follows:

(4) $${\tilde{x}}_{i}=2\times \frac{x_{i}-x_{\min}}{x_{\max}-x_{\min}}-1$$

where *x_i_* represents the *i*-th element before normalization, *x*_max_ represents the maximum values of *x_i_*, and *x*_min_ represents the minimum values of *x_i_*. Through the above normalization processing, the maximum and minimum values of the original data are limited to 1 and −1, respectively.

### 3.2. Participants

#### 3.2.1. Training Validation Experiment

Twenty healthy young adults (age = 25 ± 4 years, mass = 61.4 ± 11.3 kg, height = 167.2 ± 12.5 cm) and twenty healthy elderly adults (age = 64 ± 5 years, mass = 58.7 ± 10.7 kg, height = 165.8 ± 9.5 cm) without known lower limb musculoskeletal or neurological dysfunction participated in the training validation experiment. Written and verbal instructions of testing procedures were provided, and written consent was obtained from each subject prior to all of the experiments related to this paper. The experimental protocol was approved by the Human Ethical Review Committee of Jilin University (No. 2023-234).

A total of forty healthy adults with complete lower limb motor function participated in the training validation experiment, including twenty young adults (age = 25 ± 4 years, mass = 61.4 ± 11.3 kg, height = 167.2 ± 12.5 cm) and twenty elderly adults (age = 64 ± 5 years, mass = 58.7 ± 10.7 kg, height = 165.8 ± 9.5 cm). Prior to the experiment, each participant provided written consent, and the experimental protocol was approved by the Human Ethical Review Committee of Jilin University (No. 2023-234). After familiarizing the subjects with the experimental process, they wore the self-developed sensor system and reflective markers whose number and location were consistent with those in Gait2392 model. The trajectories of all the markers and the ground reaction forces were collected by the OMCS and force platform system, respectively. Additionally, the joint angles and angular velocities of the hip, knee, and ankle joints in the sagittal plane during human walking were collected by the wearable sensor system. For each of the 40 subjects, the data of 10 gait cycles were collected at slow speed (1.2 m/s), medium speed (1.5 m/s), and fast (1.8 m/s) speed. Finally, the data of 400 gait cycles were obtained at each of the three different speeds (10 gait cycles for each subject × 40 subjects).

Under each speed, 8 cycles of gait data were randomly selected for each subject, 320 cycles of gait data from forty subjects were selected as the training dataset, and the remaining 80 cycles of gait data were used as the validation dataset. The software used for training the neural network model in this paper was Matlab2021a, with a computer CPU model of i9-11900k and a GPU model of GTX2070ti.

#### 3.2.2. External Testing Experiment

To obtain testing experimental sample data, another three healthy young adults (age = 27 ± 6 years, mass = 66.4 ± 5.3 kg, height = 173.2 ± 5.5 cm) and three healthy elderly adults (age = 66 ± 6 years, mass = 57.4 ± 9.6 kg, height = 160.8 ± 11.5 cm) also participated in this study. For each subject, the data of two gait cycles at three different speeds were collected, and ultimately, the data of 36 gait cycles (6 gait cycles for each subject × 6 subjects) were obtained as testing dataset 1. In addition, in order to test the neural network model performance for muscle force estimation when the walking speed of the human body was outside the training dataset, the data of two gait cycles for each subject at two additional walking speeds (1.1 m/s and 1.6 m/s) were collected. Finally, another 24 gait cycles of walking data (4 gait cycles for each subject × 6 subjects) were obtained as testing dataset 2.

## 4. Results and Discussion

### 4.1. Accuracy Verification of Sensor System

**Figure 3:**
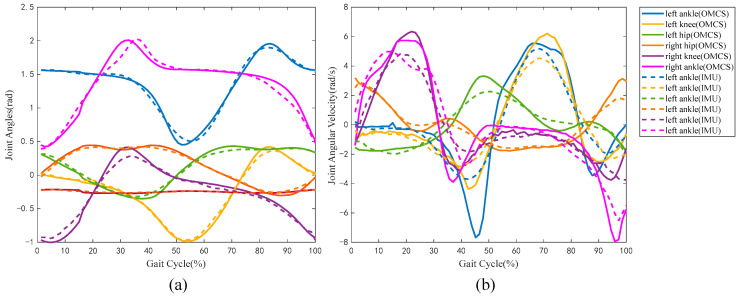
A typical group of kinematic parameters, where (**a**) represented the joint angles and (**b**) represented the joint angular velocities. The signals acquired by the self-developed sensor system are represented by the dotted lines, and those acquired by the optical motion capture system (OMCS) are represented by the solid lines.

It was necessary to verify the accuracy of the self-developed sensor system before initial use. In the training validation experiment, the data of 400 gait cycles were obtained at each of the three different speeds (10 gait cycles for each subject × 40 subjects). Ten gait cycles of data of each speed were randomly selected from the collected data, in which thirty gait cycles of data were used to verify the accuracy of the sensor system. A typical group of the kinematic parameters, including joint angles and joint angular velocities, were shown in Figure 3a,b, where the signals acquired by the self-developed sensor system were represented by the dotted lines and those acquired by the OMCS were represented by the solid lines. It can be seen from Figure 3 that the same kinematic parameters measured by the self-developed sensor system (dotted lines) and the OMCS (solid lines) showed high consistency.

The accuracy of the self-developed sensor system was evaluated by comparing it with the OMCS. As shown in Table 1, the joint angles and angular velocities measured by the sensor system were highly correlated with those measured by the OMCS (all r > 0.92 and all RMSE% < 20%), which suggested that the self-developed wearable sensor system was available for the kinematic analysis of lower limbs in gait. Of course, there were still some errors in the sensor system compared with the OMCS. Because the shape of the human body surface is irregular, it was difficult to ensure that the X-axis and Y-axis of the sensors were absolutely in the sagittal plane throughout the experiment process, although a sensor-to-segment calibration to ensure the direction of the sensor axis consistent with the direction of the limb segment was performed before the experiment. Therefore, the joint angles and angular velocities derived from the IMUs could not be absolutely accurate but with certain systematic errors and noise.

### 4.2. Overall Comparisons

**Figure 4:**
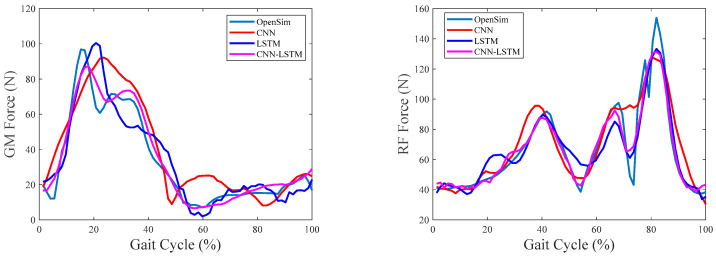

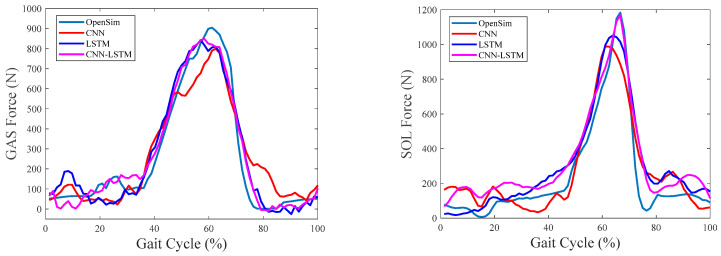
Predicted muscle forces at the slow walking speed of one gait cycle.

**Figure 5:**
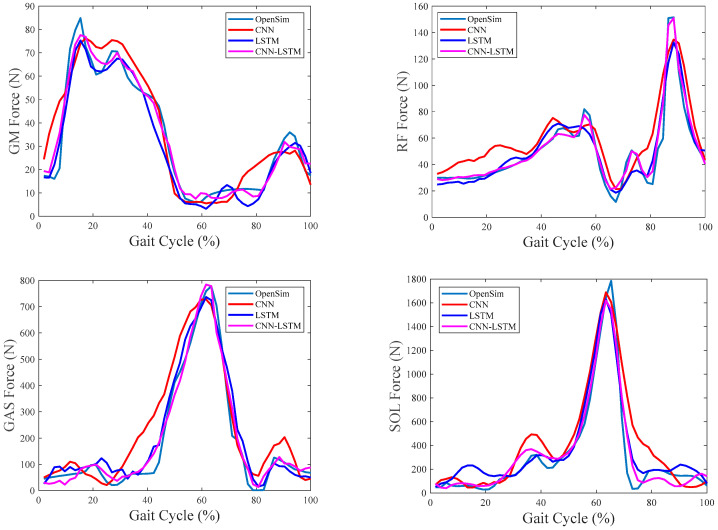
Predicted muscle forces at the medium walking speed of one gait cycle.

**Figure 6:**
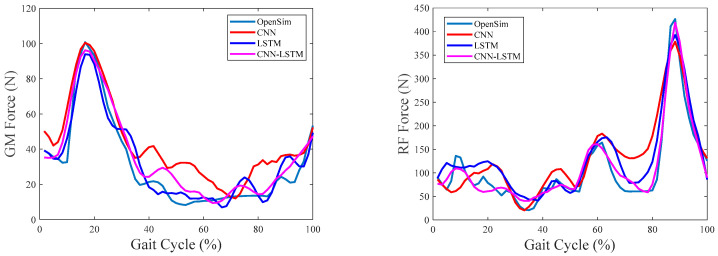

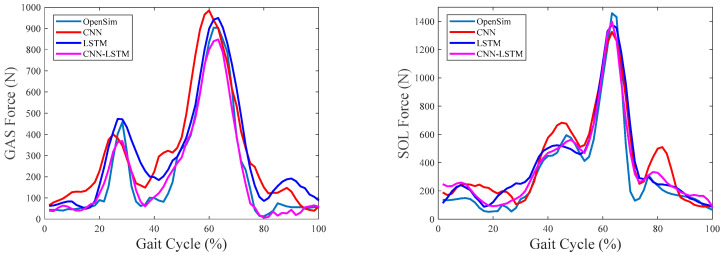
Predicted muscle forces at the fast walking speed of one gait cycle.

The overall comparisons among CNN, LSTM, and CNN–LSTM are first performed in this part. Figure 4, Figure 5 and Figure 6 show the results of the estimated muscle forces of GM, RF, GAST, and SOL using CNN, LSTM, and CNN–LSTM at slow, medium, and fast speeds, respectively. Among them, the solid green line represented the muscle forces calculated by OpenSim as the reference value, the red solid line represented the muscle force estimated by CNN, the blue solid line represented the muscle forces estimated by LSTM, and the purple–red solid line represented the muscle forces estimated by CNN–LSTM. Compared with the reference muscle forces, it could be seen that the estimation results of CNN were the worst among the three methods at any of the three walking speeds, while the estimation results of LSTM and CNN–LSTM were significantly better than CNN.

Table 2 shows the correlation coefficient between the muscle forces estimated using CNN, LSTM, and CNN–LSTM and the reference muscle forces obtained using OpenSim. It can be seen that CNN performed the worst in estimating muscle forces during slow walking, with correlation coefficients between the estimated and referenced values of the four muscles being 0.9135, 0.8943, 0.9077, and 0.8986, respectively, with an average correlation coefficient of 0.9035. However, the performances of LSTM and CNN–LSTM were significantly better than CNN, with average correlation coefficients of 0.9599 and 0.9801, respectively. The performance of CNN–LSTM was the best. When walking at medium speed, CNN also performed the worst, with an average correlation coefficient of 0.9352. Unlike at slow walking speed, LSTM performed better in estimating GAS muscle forces than CNN–LSTM at medium walking speed, with correlation coefficients of 0.9813 and 0.9809, respectively. However, CNN–LSTM still had the highest average correlation coefficient of 0.9829 when estimating muscle forces. When walking rapidly, the performance of each method was consistent with the above situation; that is, CNN performed the worst with an average correlation coefficient of 0.9098, while CNN LSTM performed the best with an average correlation coefficient of 0.9809.

In addition, it could be seen that CNN’s performance in estimating muscle forces was most affected by walking speed, with the best-performing average correlation coefficient of 0.9352 at medium speed. Compared to the worst-performing slow-speed situation, its correlation coefficient increased by 0.0317. Furthermore, the performance of LSTM and CNN–LSTM in estimating muscle forces was less affected by the walking speed, and the predicted results had smaller fluctuations. The data showed that CNN–LSTM was least affected by the walking speed, and the average change in its correlation coefficient was only 0.0028.

**Figure 7:**
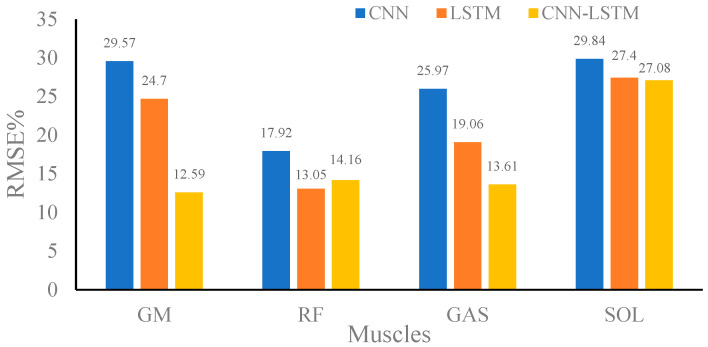
Root mean square percent (RMSE%) of the predicted muscle strength at slow speed using convolutional neural network (CNN), long-short-term memory (LSTM), and CNN–LSTM.

**Figure 8:**
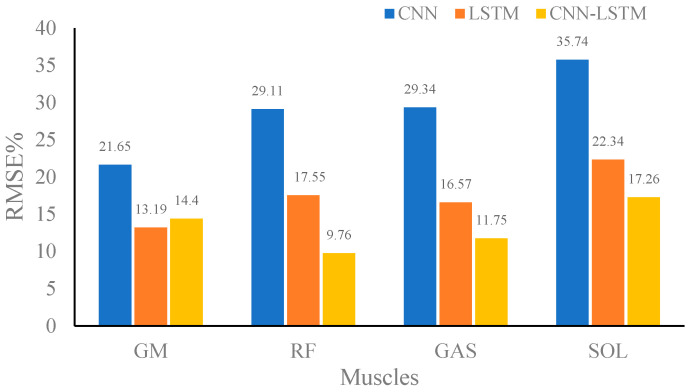
Root mean square percent (RMSE%) of the predicted muscle strength at medium speed using convolutional neural network (CNN), long-short-term memory (LSTM), and CNN–LSTM.

**Figure 9:**
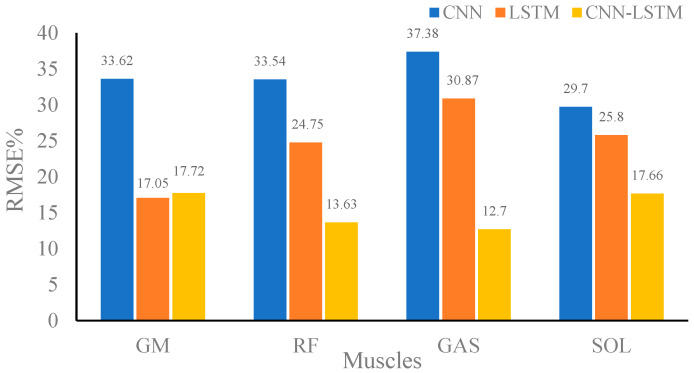
Root mean square percent (RMSE%) of the predicted muscle strength at the fast speed using convolutional neural network (CNN), long-short-term memory (LSTM), and CNN–LSTM.

Figure 7, Figure 8 and Figure 9 show the RMSE% of the estimated muscle forces of GM, RF, GAST, and SOL using CNN, LSTM, and CNN–LSTM at slow, medium, and fast speeds, respectively. It was obvious that for the four muscles, CNN had the highest RMSE% at any walking speed, with the average RMSE% being 25.83%, 28.96%, and 33.56% at slow, medium, and fast speeds, respectively. The RMSE% using LSTM or CNN–LSTM was significantly lower than the RMSE% using CNN. In most conditions, the RMSE% of CNN–LSTM was smaller than that of LSTM, indicating that CNN–LSTM performed best in estimating muscle forces, with the average RMSE% being 16.86%, 13.29%, and 15.43% at slow, medium, and fast speeds, respectively.

Based on the above analysis of the correlation coefficients and RMSE%, it could be concluded that CNN performed the worst in estimating muscle forces at any speed among CNN, LSTM, and CNN–LSTM. It suggested that simply transforming a force estimation into an image-to-image translation task without modifying the standard CNN structure was not enough. LSTM and CNN–LSTM showed significant improvement compared with CNN, whose relatively better performances had justified the use of sequential structures in force estimation tasks. CNN–LSTM performed best in predicting muscle forces and was least affected by walking speed, with the highest robustness. The results also implied that, to some extent, a higher-level representation of input data can indeed improve the prediction accuracy.

### 4.3. Evaluation of Intrasession Scenario

Furthermore, in order to verify the comprehensive performance of CNN–LSTM in estimating muscle forces, all the obtained data were fused into a whole dataset, which included the data of 1200 gait cycles (10 gait cycles at each walking speed for each subject performed by 40 subjects at 3 different speeds). We took the data of 960 gait cycles for neural network training, and the remaining data were used for subsequent validation.

Table 3 shows the correlation coefficients and RMSE% for estimating muscle forces using CNN, LSTM, and CNN–LSTM. It can be seen that CNN–LSTM performed the best with an average correlation coefficient and RMSE% of 0.9807 and 15.14% between the estimated and referenced values.

### 4.4. Evaluation of Intersession Scenario

**Figure 10:**
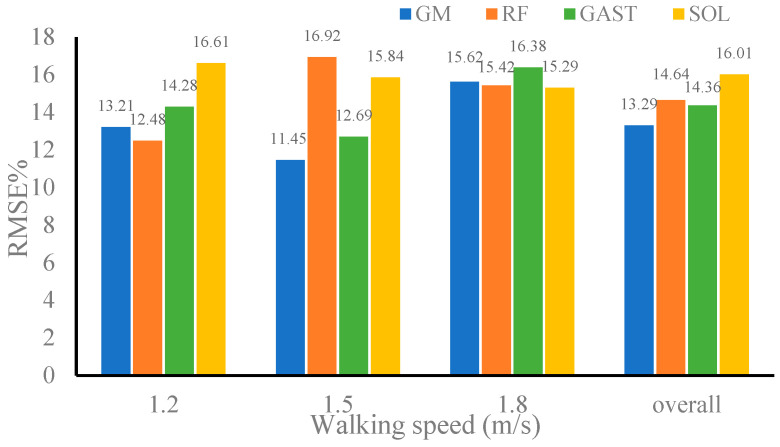
RMSE% of muscle forces estimated by convolutional neural network–long-short-term memory (CNN–LSTM) when using dataset 1.

**Figure 11:**
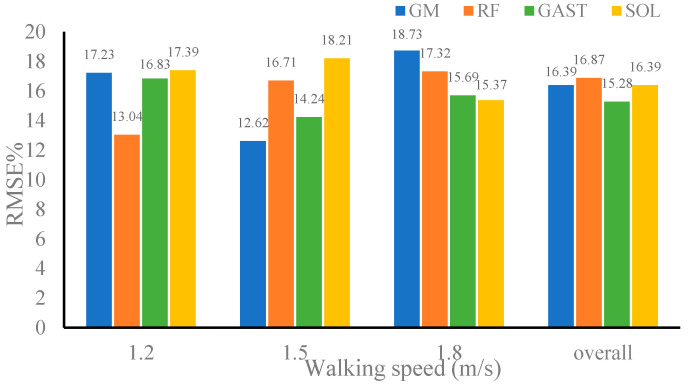
RMSE% of muscle forces estimated by convolutional neural network–long-short-term memory (CNN–LSTM) when using dataset 2.

Table 4 shows the correlation coefficients between the estimated and referenced values of the muscle forces of the GM, RF, GAST, and SOL using CNN–LSTM for validation datasets 1 and 2. Figure 10 and Figure 11 represent the RMSE% of muscle forces estimated by CNN–LSTM using validation dataset 1 and validation dataset 2, respectively. It can be seen that when the gait data of the three walking speeds in validation dataset 1 were used as inputs to CNN–LSTM, the correlation coefficients between the estimated and referenced muscle forces of the four muscles were not less than 0.9720, and the RMSE% was not greater than 16.92%. Meanwhile, the gait data of the three walking speeds in validation dataset 1 were randomly combined as inputs to CNN–LSTM, and the correlation coefficients of the four muscles were 0.9793, 0.9846, 0.9719, and 0.9802, with an average correlation coefficient of 0.9790. The RMSE% of the four muscles was 13.29%, 14.64%, 14.36%, and 16.01%, respectively, with the average RMSE% being 14.58%. The performance of CNN–LSTM in estimating muscle forces using validation dataset 1 showed only a slight decrease compared to that using the training dataset, but it did not affect the usability of the model. The above results indicated that the performance of the CNN–LSTM model proposed in this paper in muscle forces estimation was minimally affected by individual differences and had strong universality.

It also could be seen that when the gait data at two walking speeds in validation dataset 2 were used as inputs to CNN–LSTM, the correlation coefficients between the estimated and referenced muscle forces of the four muscles were not less than 0.9680, and the RMSE% was not greater than 18.73%. Meanwhile, the gait data of the two walking speeds in validation dataset 2 were randomly combined as inputs to CNN–LSTM, and the correlation coefficients of the four muscles were 0.9728, 0.9669, 0.9738, and 0.9733 with an average correlation coefficient of 0.9717. The RMSE% of the four muscles was 16.39%, 16.87%, 15.28%, and 16.39%, respectively, with the average RMSE% being 16.23%. The above results indicated that there was no significant difference in the average correlation coefficient of CNN–LSTM in estimating muscle forces when using validation dataset 2 or validation dataset 1, respectively. The same conclusion was reached for the analysis of RMSE%. Although the training dataset in this paper did not include gait data at 1.1 m/s and 1.6 m/s, CNN–LSTM still performed well in muscle force estimation at these two speeds. This fully indicated that the CNN–LSTM proposed in this article had good robustness and generalization in estimating muscle forces.

### 4.5. Advantages and Limitations of the CNN–LSTM

This article has proposed a muscle forces estimation method based on CNN–LSTM and evaluated its performance. The results showed that the model performed well in estimating muscle forces. Compared to existing methods, the proposed method has significant advantages. The traditional muscle force calculation methods mainly include static optimization algorithms and algorithms based on sEMG signals. When using static optimization to calculate muscle forces, it is necessary to first calculate joint moments and then select appropriate optimization objectives and functions. The optimization process itself requires high computational power, and as the number of muscles involved in the optimization process increases, the complexity of the optimization process also increases. When using muscle force calculation methods based on sEMG signals, it is often necessary to determine some physiological parameters. For example, when using the Hill model to calculate muscle forces, it is necessary to determine physiological parameters, such as the pennation angle, muscle length, and muscle activation of the muscle; however, these parameters are difficult to directly measure, which limits the application of muscle force calculation methods. Compared to the above two methods, the muscle forces estimation method based on CNN–LSTM proposed in this article is more convenient and faster. The method only requires collecting gait data during walking to obtain muscle forces and has potential application prospects in gait evaluation and the development of rehabilitation equipment. Of course, the method proposed in this article also has limitations. This article only studied the performance of CNN–LSTM in estimating muscle forces during straight-line walking and did not investigate the applicability of the model to other activities, such as running and jumping. In future work, this will become our research focus.

## 5. Conclusions

This paper proposed a novel muscle forces estimation method based on the CNN–LSTM neural network and the self-developed wearable sensor system. The CNN–LSTM model encompassed the advantages of both CNN and LSTM—CNN is good at extracting features, and LSTM is good at processing time sequence data. The experiment results showed that CNN–LSTM had good robustness and generalization compared to the standard CNN and LSTM. Compared to the method for calculating muscle forces using OpenSim, the method proposed in this paper can not only ensure a satisfactory accuracy in estimating muscle forces but also achieve an effective algorithm for estimating muscle forces through wearable and convenient experimental devices. There were still some limitations to this article, which only verified the performance of the CNN–LSTM in estimating muscle forces during forward straight walking without considering the performance of the model during turns, stairs, and running. In future work, we will evaluate the performance of the CNN–LSTM in muscle force estimation under different human motions.

## Figures and Tables

**Table 1:** Analysis and comparison of joint angles and joint angular velocities derived from inertial measurement units (IMUs) and optical motion capture system (OMCS) in gait.

Kinematics Parameters	RMSE%	r
Left ankle joint angle (rad)	13.03	0.969
Left knee joint angle (rad)	10.36	0.981
Left hip joint angle (rad)	9.51	0.979
Right hip joint angle (rad)	10.84	0.982
Right knee joint angle (rad)	11.06	0.969
Right ankle joint angle (rad)	14.31	0.971
Left ankle joint angular velocity (rad/s)	18.72	0.932
Left knee joint angular velocity (rad/s)	13.87	0.924
Left hip joint angular velocity (rad/s)	19.31	0.926
Right hip joint angular velocity (rad/s)	16.42	0.947
Right knee joint angular velocity (rad/s)	17.14	0.953
Right ankle joint angular velocity (rad/s)	16.77	0.921

**Table 2:** The correlation coefficients among the muscle forces estimated using convolutional neural network (CNN), long-short-term memory (LSTM), and CNN–LSTM and the reference muscle forces obtained using OpenSim.

Gait Speed	Muscle	CNN	LSTM	CNN–LSTM
Slow speed	GM	0.9135	0.9608	0.9798
	RF	0.8943	0.9514	0.9749
	GAS	0.9077	0.9681	0.9841
	SOL	0.8986	0.9591	0.9816
	Average	0.9035	0.9599	0.9801
Normal speed	GM	0.9388	0.9768	0.9838
	RF	0.9186	0.9336	0.9814
	GAS	0.9399	0.9813	0.9809
	SOL	0.9434	0.9704	0.9776
	Average	0.9352	0.9655	0.9829
Fast speed	GM	0.9282	0.9665	0.9765
	RF	0.8855	0.9488	0.9762
	GAS	0.9055	0.9720	0.9877
	SOL	0.9199	0.9555	0.9832
	Average	0.9098	0.9607	0.9809

**Table 3:** Correlation coefficients and root mean square percent (RMSE%) for predicting muscle forces using convolutional neural network (CNN), long-short-term memory (LSTM), and CNN–LSTM.

	Muscle	CNN	LSTM	CNN–LSTM
Pearson’s correlation coefficient (*r*)	GM	0.9174	0.9364	0.9776
	RF	0.9017	0.9488	0.9798
	GAS	0.9217	0.9674	0.9848
	SOL	0.9329	0.9604	0.9808
	Average	0.9184	0.9532	0.9807
Percentage root mean square error (RMSE%)	GM	27.37	20.19	12.54
	RF	32.64	14.83	15.01
	GAS	29.87	22.31	14.23
	SOL	30.69	21.22	18.78
	Average	30.15	19.64	15.14

**Table 4:** Correlation coefficients of convolutional neural network–long-short-term memory (CNN–LSTM) estimation of muscle forces using validation dataset 1 and validation dataset 2, respectively.

	Gait Speed	GM	RF	GAS	SOL
Dataset 1	1.2 m/s	0.9765	0.9823	0.9689	0.9758
	1.5 m/s	0.9828	0.9874	0.9808	0.9783
	1.8 m/s	0.9804	0.9768	0.9728	0.9806
	Overall	0.9793	0.9846	0.9719	0.9802
Dataset 2	1.1 m/s	0.9737	0.9721	0.9763	0.9803
	1.6 m/s	0.9698	0.9687	0.9742	0.9763
	Overall	0.9728	0.9669	0.9738	0.9733

## Data Availability

Data is unavailable due to privacy or ethical restrictions.
